# Structure and Magnetism of Co_2_Ge Nanoparticles

**DOI:** 10.3390/nano9101371

**Published:** 2019-09-25

**Authors:** Onur Tosun, Frank M. Abel, Balamurugan Balasubramanian, Ralph Skomski, David J. Sellmyer, George C. Hadjipanayis

**Affiliations:** 1 Department of Physics and Astronomy, University of Delaware, Newark, DE 19711, USA; onurt@udel.edu (O.T.); fabel@udel.edu (F.M.A.); 2 Department of Physics and Astronomy, University of Nebraska, Lincoln, NE 68588, USA; balamurugan@unl.edu (B.B.); rskomski@neb.rr.com (R.S.); dsellmyer@unl.edu (D.J.S.); 3 Nebraska Center for Materials and Nanoscience, University of Nebraska, Lincoln, NE 68588, USA

**Keywords:** magnetic nanoparticles, cluster deposition, Curie temperature, magnetocrystalline anisotropy

## Abstract

The structural and magnetic properties of Co_2_Ge nanoparticles (NPs) prepared by the cluster-beam deposition (CBD) technique have been investigated. As-made particles with an average size of 5.5 nm exhibit a mixture of hexagonal and orthorhombic crystal structures. Thermomagnetic measurements showed that the as-made particles are superparamagnetic at room temperature with a blocking temperature (*T_B_*) of 20 K. When the particles are annealed at 823 K for 12 h, their size is increased to 13 nm and they develop a new orthorhombic crystal structure, with a Curie temperature (*T_C_*) of 815 K. This is drastically different from bulk, which are ferromagnetic at cryogenic temperatures only. X-ray diffraction (XRD) measurements suggest the formation of a new Co-rich orthorhombic phase (OP) with slightly increased c/a ratio in the annealed particles and this is believed to be the reason for the drastic change in their magnetic properties.

## 1. Introduction

Magnetic nanoparticles (NPs) have unique and interesting properties, which are both scientifically important and attractive for numerous advanced technologies, due to both size and surface effects. These effects have been attributed to changes in the electronic structure, spin structure and spin polarization of the NPs because of the large fraction of surface atoms [1,2,3,4,5,6]. Numerous studies have shown the importance of magnetic NPs with potential applications in high-temperature permanent magnets [7,8,9], catalysis [10,11,12] and nanoelectronics [13,14]. 

Our previous studies have shown how new materials and spin structures can be created through the judicious use of non-equilibrium synthesis, element substitutions, and nanostructuring. We have shown how atomic-scale exchange phenomena can be controlled and exploited in nanoscale itinerant magnets to substantially improve their magnetic properties. A unique nanoscale moment-enhancement in Co_2_Si nanoclusters as compared to that of bulk was reported by Balasubramanian et al. [15]. A larger magnetization and higher Curie temperature (*T_C_*) have also been observed in Fe_5_Si_3_ clusters [16]. We have also managed to synthesize hexagonal Co_3_Si clusters, which showed large coercivities even though the material has a planar anisotropy in bulk [17].

In this study, our research is focused on nanoclusters of Co-rich Co-Ge alloys. Most of the studies so far in this system have been focused on bulk alloys with very few studies on mechanically alloyed samples and they all suggest the existence of two phases, a Ni_2_In-type high temperature hexagonal phase (HP) with a Pearson symbol of hP6 and space group of P6_3_/mmc and a Co_2_Si-type low temperature OP with a Pearson symbol of oP12 and space group 62 [18]. Zhou et al. [18] found that their magnetic properties depend strongly on crystalline ordering and determined the *T_C_* of the HP and OP Co_2_Ge to be 6 and 46.4 K, respectively. Tosun et al. [19], on the other hand, showed that the structural and magnetic properties of melt-spun Co–Ge alloys depend on the Co content, and their solidification rate and annealing conditions; they found the *T_C_* of the HP and OP in the as-made Co_66_Ge_34_ alloys to be 105 and 425 K, respectively. To our knowledge, there is not much information available on the synthesis and characterization of Co–Ge at the nanoscale. In this work, we have synthesized isolated Co_2_Ge NPs by the cluster-beam deposition method and studied their structural and magnetic properties in the as-made and annealed states. 

## 2. Materials and Methods 

The Co_2_Ge NPs were fabricated using the cluster-beam deposition (CBD) method previously described by Tosun et al. [5]. The cluster-gun is a magnetron sputtering gun in an aggregation chamber where a high Argon (Ar) pressure is applied typically in the range of 0.07–0.7 kPa. A compacted powder target, which was made by arc-melting appropriate amounts of high purity cobalt (99.9999%) and germanium (99.9999%) (Alfa Aesar Ltd., Ward Hill, MA, USA) elements, was sputtered at a high DC power of 60 W under a high-purity (99.9999%) Ar (Keen Compressed Gas Co., Wilmington, DE, USA) pressure of 0.13 kPa. The particles formed are deposited as dense films on 500 μm-thick Si wafer (Nova Electronic Materials LLC, Flower Mound, TX, USA) for X-ray diffraction (XRD) and magnetic measurements and on C-coated Cu grids (Electron Microscopy Sciences Inc., Hatfield, PA, USA) for transmission electron microscopy (TEM) studies. The samples were coated with a silver (Alfa Aesar Ltd., Ward Hill, MA, USA) capping layer using magnetron sputtering at a DC power of 10 W in order to prevent oxidation and they were annealed in sealed quartz tubes at 823 K for 12 h. Diamagnetic contributions due to the Si wafer, Ag-capping-layer, and Kapton tape (Shercon Inc., Santa Fe Springs, CA, USA) used for magnetometry measurements were determined properly and subtracted from the raw data. A JEOL JSM 6330F Scanning Electron Microscope (SEM; JEOL Ltd., Akishima, Tokyo, Japan) was used to perform the compositional analysis and to determine the volume fraction of the magnetic phases. The structural analyses were performed in a Rigaku Ultima IV X-ray diffractometer (Rigaku Corp., Akishima, Tokyo, Japan) using Cu Kα radiation with a wavelength of 1.540 Å, and a JEOL JEM-3010 Transmission Electron Microscope (TEM; JEOL Ltd., Akishima, Tokyo, Japan) operating at 300 kV. Elemental mapping measurements on the particles were performed with FEI Tecnai Osiris Scanning Transmission Electron Microscope (STEM; FEI Ltd., Hillsboro, OR, USA) The magnetometry measurements of the samples were carried out with a Quantum Design Versa Lab Vibrating Sample Magnetometer (VSM; Quantum Design Inc., San Diego, CA, USA) and a physical property measurement system (PPMS; Quantum Design Inc., San Diego, CA, USA).

## 3. Results and Discussion

### 3.1. Crystal Structure and Microstructure Measurements

**Figure 1:**
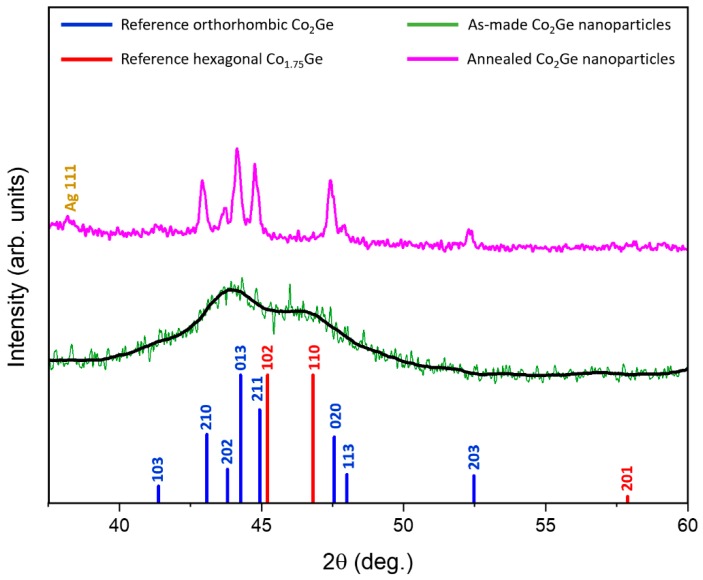
X-ray diffraction (XRD) patterns of the as-made and annealed Co_2_Ge nanoparticles (NPs). The XRD reflections of the orthorhombic phase (OP) are shown with the blue lines [20] and the hexagonal phase (HP) are shown with red lines [21].

**Figure 2:**
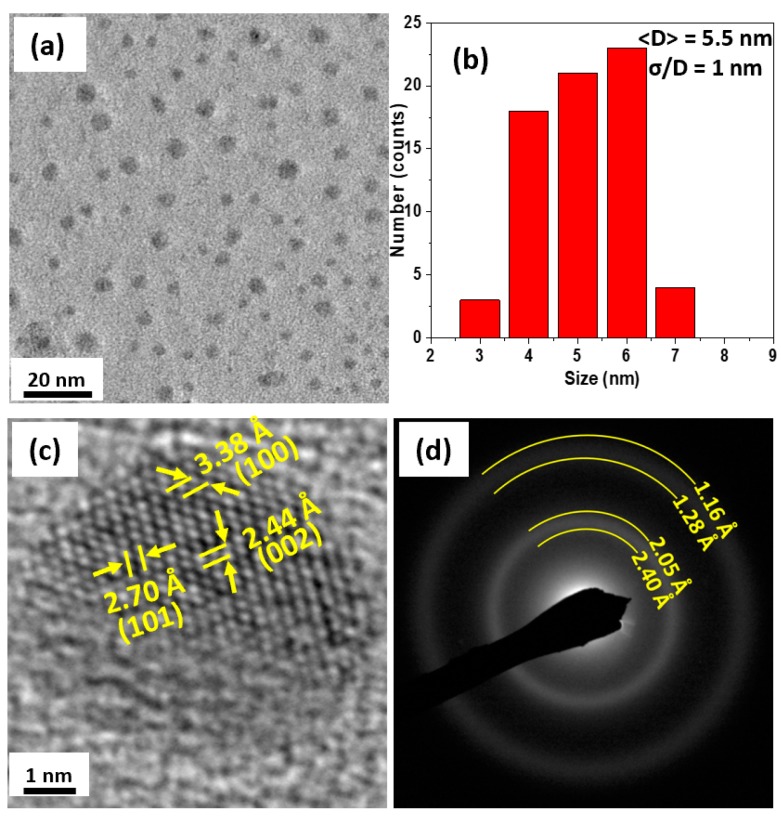
Transmission electron microscope (TEM) studies on the as-made NPs. Figures (**a**–**d**) show the particles observed by the TEM, their size distribution, the high resolution TEM (HR-TEM) and selected area diffraction (SAD) images of the as-made NPs, respectively.

Energy dispersive spectroscopy (EDS) measurements show that the NPs had a composition similar to the target Co_2_Ge composition. Figure 1 shows the XRD data of the as-made and annealed NPs as well as the reference XRD spectra (lines) of the bulk HP and OP. It can be seen from Figure 1 that the XRD peaks of the as-made NPs were broad suggesting that the particles were very small. Figure 2 shows the TEM studies on the as-made NPs. As can be seen from the TEM micrographs in Figure 2c the particles show a mixture of crystalline and highly disordered/amorphous regions. 

The lattice parameters and the volume fractions of the phases present in the as-made and annealed samples were determined by fitting the XRD data to the reference data obtained by P. K. Panday et al. [20] (*a* = 5.020 Å, *b* = 3.820 Å, and *c* = 7.260 Å) and M. Ellner et al. [21] (*a* = 3.914 Å, and *c* = 5.004 Å) using a software reported in reference [22]. The volume fractions of the phases present in the as-made sample were determined by Rietveld refinement analysis, which suggests that the as-made NPs consist of 27 vol. % HP and 73 vol. % OP crystal structures, respectively. The amorphous feature of the as-made NPs was not considered in the Rietveld analysis. Instead, the XRD reflections of both the OP and HP were broadened for the best fit.

Our TEM and high-resolution TEM (HRTEM) data in Figure 2a–d show that the as-made NPs have an average size of 5.5 nm and they are partially crystalline and highly disordered/amorphous (Figure 2c). Moreover, the diffraction rings obtained by selected-area-diffraction (SAD) measurements shown in Figure 2d are broad, which is another indication that the as-made NPs are very small and not well crystalline. The broad SAD rings make it difficult to measure the *d*-spacing values of crystallographic reflections of the phases present. 

Instead, the *d*-spacing values of the borders of the broad SAD rings were measured and the results suggest that the as-made sample could be a mixture of the OP and HP. The *d*-spacing values of the lattice fringes of the crystalline part of the NP shown in Figure 2c are fitted to the (100), (101), and (002) reflections of the HP.

**Figure 3:**
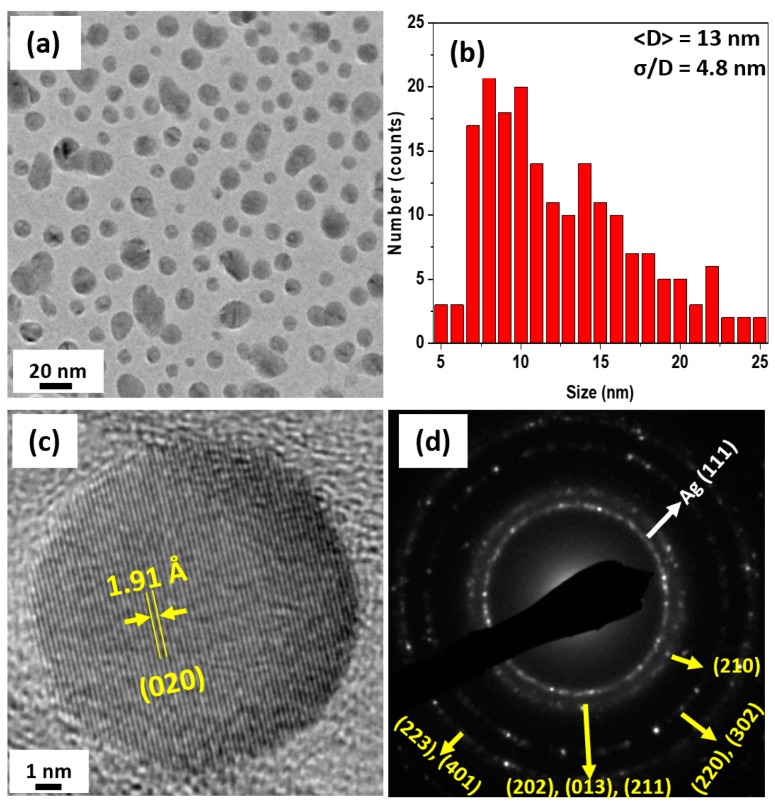
TEM studies on the annealed NPs. Figures (**a**–**d**) show the particles observed by the TEM, their size distribution, the HR-TEM and SAD images of the annealed NPs, respectively.

The XRD pattern of annealed particles shows sharper peaks, which are characteristic of larger and well crystalline particles. According to our Rietveld refinement analysis, the NPs annealed at 823 K have a nearly single-phase OP (98 vol. %). The results of TEM observations are shown in Figure 3. Figure 3a,b show that the annealed NPs had an average size of 13 nm with a very broad size distribution. The HR-TEM image (Figure 3c) clearly shows that the annealed NPs were fully crystalline with very sharp lattice fringes, which were fitted to the (020) reflection of the OP. 

The SAD rings shown in Figure 3d were sharp and stronger and can be fitted to the OP structure. It is interesting to note that the c/a ratio of the new OP phase is higher and close to the value reported by Tosun et al. [19] on melt-spun samples with similar composition. The volume fractions and lattice parameters of the OP and HP in the as-made and annealed NPs are shown in Table 1. The error in the lattice parameters of the as-made sample is in the second decimal, which is rather large. 

### 3.2. Magnetic Properties

**Figure 4:**
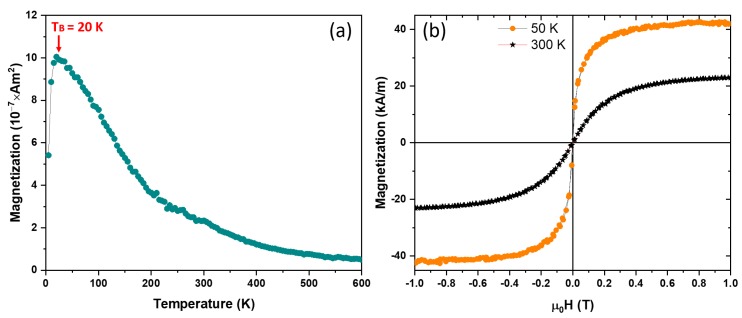
Magnetic properties of the as-made NPs: (**a**) Magnetization (M) vs. temperature (T) data of the as-made NPs; and (**b**) hysteresis loops of the as-made NPs at 300 and 50 K.

The as-made NPs were first zero field cooled (ZFC) to 50 K and then a 50 mT magnetic field was applied and the magnetization versus temperature (M vs. T) was measured. These thermomagnetic measurements (M vs. T) in Figure 4a show that the as-made NPs were superparamagnetic with a blocking temperature (*T_B_*) of 20 K. The saturation magnetization (*M_s_*) values of the as-made NPs at 50 and 300 K were determined by fitting the data to the law-of-approach to saturation [23,24] and they were determined to be 43 and 23 kA/m, respectively and no coercivity (*H_c_*) was observed at temperatures down to 50 K as shown in Figure 4b. 

**Figure 5:**
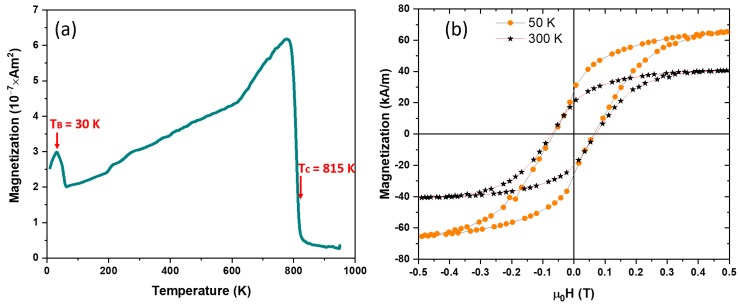
Magnetic properties of the annealed NPs: (**a**) M vs. T data of the annealed NPs; and (**b**) hysteresis loops of the annealed NPs at 300 and 50 K.

Thermomagnetic measurements on the annealed NPs, which were 98% OP, are shown in Figure 5a. These NPs were ferromagnetic at room temperature with a very high *T_C_* of 815 K. This behavior was dramatically different when compared to the bulk values showing a ferromagnetic ordering only at cryogenic temperatures (*T_C_* of the bulk HP and OP Co_2_Ge were 6 and 46.4 K, respectively [18]). This new OP has been observed also in annealed melt-spun ribbons [19]. The small peak at low temperatures (Figure 5a) could be attributed to *T_B_* of 30 K for particles with sizes smaller than 13 nm and bigger than 5.5 nm (in comparison to the as-made NPs). The gradual increase in the magnetization with increasing temperature as shown in Figure 5a was due to the Hopkinson effect. The sharp increase in magnetization starting at around 670 K was due to the increase of the amount of the ‘new’ OP. The *M_s_* and *H_c_* values of the annealed NPs were ~70 kA/m and 38 kA/m, and 65 mT and 70 mT at 50 K and 300 K, respectively, as shown in Figure 5b. These values were much larger than those reported in bulk samples.

**Figure 6:**
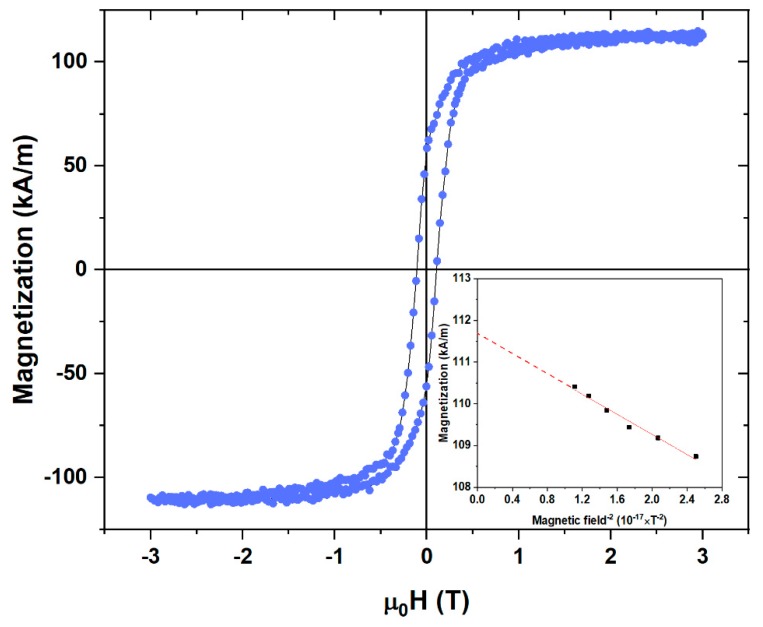
Hysteresis loop of the annealed NPs at 5 K.

A hysteresis loop measurement for the annealed particles was performed at 5 K as shown in Figure 6. The enhanced values of *M_s_* = 112 kA/m and *H_c_* = 115 mT at 5 K could be attributed to the fact that all the particles were now thermally blocked below their *T_B_* = 30 K. The magnetization data were fitted to the law-of-approach to saturation to determine their anisotropy, K, using the expression *M* = *M_s_* (1-A/H^2^) + χH for uniaxial isotropic particles [23,24] where A = (4/15)(*K^2^*/*M_s_^2^*), χ is the high-field susceptibility, and *K* is the effective anisotropy that includes surface anisotropy. This analysis is shown in the inset of Figure 6 and it yields a value of *K* = 60 kJ/m^3^. 

**Figure 7:**
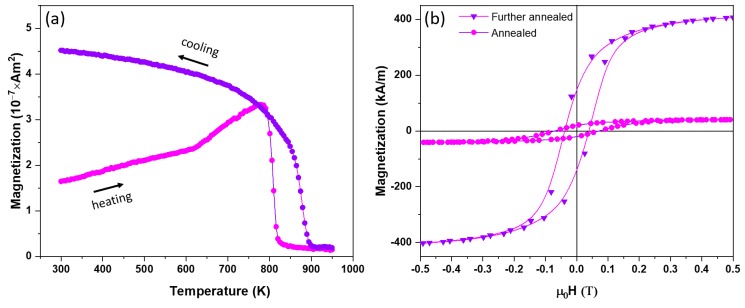
Magnetic properties of the annealed and further annealed NPs: (**a**) M vs. T data of the annealed NPs; and (**b**) hysteresis loops of the annealed and further annealed NPs at 300 K.

**Figure 8:**
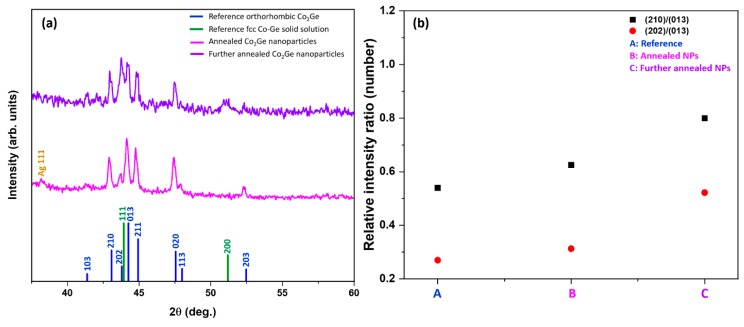
Structure of the annealed and further annealed NPS: (**a**) XRD data of the annealed and further annealed NPs as well as the reference XRD lines; and (**b**) the relative intensity ratios of (210)/(013) and (202)/(013) of XRD lines of the OP in all the annealed samples and reference lines.

In order to understand better the much larger *M_s_* and *T_C_* values of the NPs as compared to those of bulk samples and compare the behavior of Co_2_Ge NPs with our previous work on the melt-spun alloys [19], the annealed NPs were further heated during a thermomagnetic measurement as shown in Figure 7a to a temperature of 950 K. The further annealed particles were found to have a slightly increased *T_C_* = 870 K, four times increased *M_s_* = 478 kA/m but decreased *H_c_* = 40 mT as shown in Figure 7b. The room temperature *K* value of the further annealed sample was 25 kJ/m^3^. The drastic increase in magnetization and decrease in the *H_c_* value might be explained by the presence of the fcc Co–Ge solid solution because it had a higher *M_s_* and a lower anisotropy because it was cubic. XRD measurements of the further annealed sample showed that there were notable increases in the relative intensities of the (210) and (202) peaks as well as the emergence of (111) and (200) XRD reflections of fcc Co–Ge solid solution as shown in Figure 8a. The XRD data of the as-made and annealed NPs as well as the reference lines are shown in Figure 8a for comparison. The relative intensity ratios of (210)/(013) and (202)/(013) of XRD lines of the OP in all the annealed samples and reference lines are shown in Figure 8b. Our Rietveld analysis revealed that when the occupancy number of Co atoms in the Co lattice sites was increased at the expense of Ge in the Ge sites, the intensity ratios of (210)/(013) and (202)/(013) of the OP increase. The Co–Co exchange interaction increases due to the Co atoms replacing the Ge atoms in the Ge sites. We believe that the increase in the Co­–Co exchange interaction leads to the increase in both magnetization and *T_C_* values in the NPs with the new OP.

**Figure 9:**
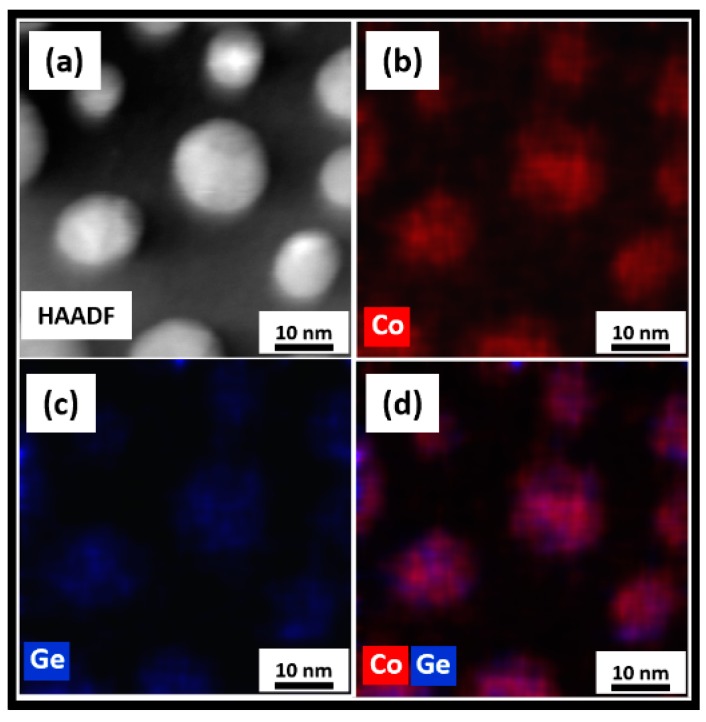
Scanning transmission electron microscope (STEM) studies on the as-made Co_2_Ge NPs: (**a**) A high-angle annular dark-field (HAADF) image and the corresponding energy dispersive spectroscopy (EDS) elemental color mappings showing; (**b**) Co; (**c**) Ge; and (**d**) Co and Ge distributions.

**Figure 10:**
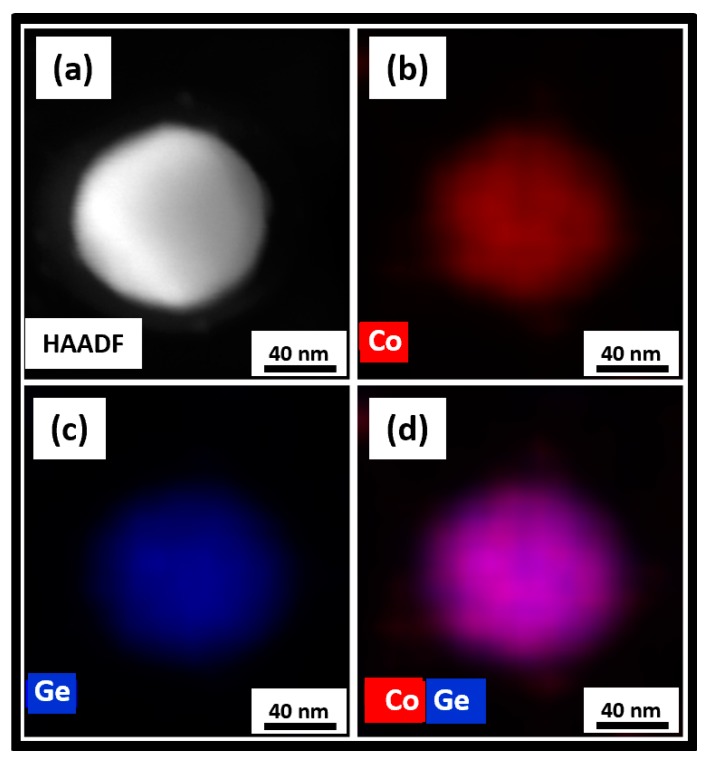
STEM studies on the annealed Co_2_Ge NPs: (**a**) A HAADF image and the corresponding EDS elemental color mappings showing; (**b**) Co; (**c**) Ge; and (**d**) Co and Ge distributions.

**Figure 11:**
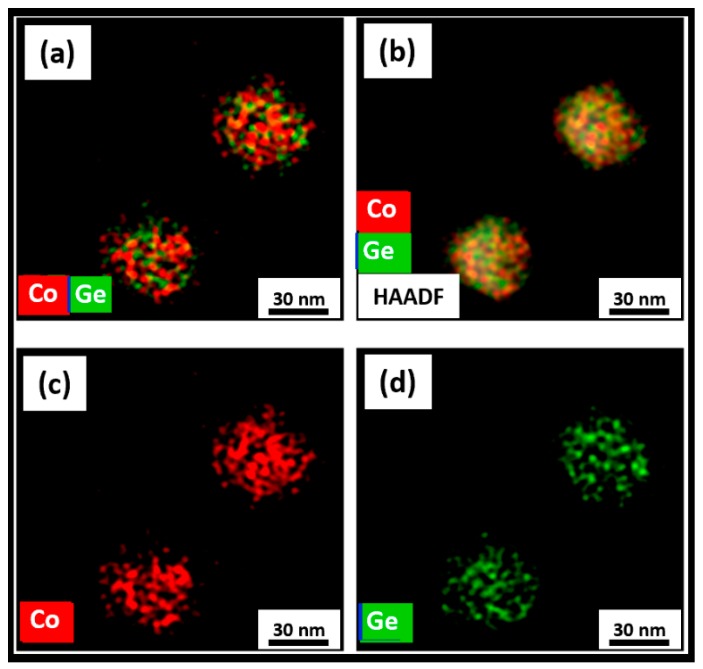
STEM studies on the further annealed Co_2_Ge NPs and EDS elemental color mappings showing: (**a**) Co and Ge distributions; (**b**) Co and Ge distributions with a HAADF image; (**c**) Co; and (**d**) Ge.

The STEM analyses shown in Figure 9, Figure 10 and Figure 11 summarized the microstructure of the as-made, annealed, and further annealed NPs. Figure 9 makes it clear that the magnetic properties of the as-made NPs are due to their disordered crystal structure. It can be seen from Figure 9d that Co and Ge are not uniformly distributed inside the NPs. The small Co clusters of 3–5 nm formed in the NPs explained the superparamagnetism observed. This feature of the as-made NPs was also reflected in their XRD and TEM data. However, the Co and Ge atoms were uniformly distributed over the annealed NPs shown in Figure 10 where no separate Co or Ge regions were observed. This microstructure corresponded to the NPs with the new OP that showed the very high *T_C_*. The STEM image of the further annealed NPs to 950 K is shown in Figure 11. It is obvious from Figure 11a that there were clear phase segregations in these NPs with Co-rich regions of 15–20 nm, which could give rise to the high *T_C_* of 870 K measured. 

## 4. Conclusions 

In summary, we synthesized Co_2_Ge nanoparticles with the orthorhombic crystal structure and found interesting magnetic properties, which were drastically different from bulk. Unlike bulk Co_2_Ge, the as-made Co_2_Ge nanoparticles were superparamagnetic at room temperature due to their small particle size. However, the annealed nanoparticles were ferromagnetic with a high Curie temperature, *T_C_* = 815 K and appreciable coercivity, *H_c_* = 70 mT. These enhanced properties might be attributed to a newly formed orthorhombic phase with a composition richer in Co and with slightly increased c/a ratio. Further heating leads to an increase in *T_C_* to 870 K. The magnetic and structural properties were consistent with elemental mapping observations. The as-made particles show that Co and Ge atoms were located separately over the particles. However, the nanoparticles annealed at 823 K show a uniform distribution of Co and Ge atoms suggesting a single phase. The further annealed nanoparticles show Co-segregation at the particle surface and this could explain the small increase in *T_C_* to 870 K.

## Figures and Tables

**Table 1:** The volume fractions and lattice parameters of the hexagonal phase (HP) and orthorhombic phase (OP) in the as-made and annealed nanoparticles (NPs).

Sample	HP (%)	OP (%)	HP Lattice Parameters (Å)	HP c/a	OP Lattice Parameters (Å)	OP c/a	OP c/b
As-made	27	73	a = 3.8777 c = 4.9748	1.28 (1)	a = 5.0134 b = 3.8209 c = 7.2586	1.43 (1)	1.86 (1)
Annealed	2	98	--------	--------	a = 5.0279 b = 3.8235 c = 7.2504	1.4420	1.8962
